# Post-pandemic influenza A/H1N1pdm09 is associated with more severe outcomes than A/H3N2 and other respiratory viruses in adult hospitalisations

**DOI:** 10.1017/S095026881900195X

**Published:** 2019-11-28

**Authors:** C. A. Minney-Smith, L. A. Selvey, A. Levy, D. W. Smith

**Affiliations:** 1 School of Public Health, Curtin University, Bentley, Western Australia, Australia; 2 PathWest Laboratory Medicine WA, Nedlands, Western Australia, Australia; 3 School of Public Health, The University of Queensland, Herston, Queensland, Australia; 4 School of Biomedical Sciences, University of Western Australia, Nedlands, Australia

**Keywords:** A/H1N1pdm09, A/H3N2, hospitalisation, influenza, respiratory viruses

## Abstract

This study compares the frequency and severity of influenza A/H1N1pdm09 (A/H1), influenza A/H3N2 (A/H3) and other respiratory virus infections in hospitalised patients. Data from 17 332 adult hospitalised patients admitted to Sir Charles Gairdner Hospital, Perth, Western Australia, with a respiratory illness between 2012 and 2015 were linked with data containing reverse transcription polymerase chain reaction results for respiratory viruses including A/H1, A/H3, influenza B, human metapneumovirus, respiratory syncytial virus and parainfluenza. Of these, 1753 (10.1%) had test results. Multivariable regression analyses were conducted to compare the viruses for clinical outcomes including ICU admission, ventilation, pneumonia, length of stay and death. Patients with A/H1 were more likely to experience severe outcomes such as ICU admission (OR 2.5, 95% CI 1.2–5.5, *P* = 0.016), pneumonia (OR 3.0, 95% CI 1.6–5.7, *P* < 0.001) and lower risk of discharge from hospital (indicating longer lengths of hospitalisation; HR 0.64 95% CI 0.47–0.88, *P* = 0.005), than patients with A/H3. Patients with a non-influenza respiratory virus were less likely to experience severe clinical outcomes than patients with A/H1, however, had similar likelihood when compared to patients with A/H3. Patients hospitalised with A/H1 had higher odds of severe outcomes than patients with A/H3 or other respiratory viruses. Knowledge of circulating influenza strains is important for healthcare preparedness.

## Introduction

Influenza infections have a large impact on human morbidity and mortality each year [1]. This results in a substantial additional burden of hospital admissions each winter, both internationally and within Australia [2]. The severity and clinical impact of an influenza season varies from year to year and depends on a number of factors such as vaccination efficacy and circulating influenza strain. Early evidence analysing the 2009 H1N1 pandemic suggested that influenza A/H1N1pdm09 (A/H1) may have more severe clinical impacts than other seasonal influenzas [3, 4]. This evidence continues to be supported in the post-pandemic years with several studies showing that patients with A/H1 have an increased risk of severe disease compared to patients with influenza A/H3N2 (A/H3) [5, 6].

In addition to influenza, there is also growing evidence of the importance of other viruses causing respiratory illnesses resulting in hospitalization [7, 8] and for which there are currently no vaccines and few treatment options available [9, 10]. Prominent among these are respiratory syncytial virus (RSV), human rhinoviruses, human coronaviruses, human metapneumovirus (hMPV) and parainfluenza viruses (PIV) on hospitalisation. Most studies have been conducted in children, but it appears that these other viruses are likely to be of similar importance in adults [7, 11–14].

Describing the impact of specific influenza subtypes, as well as non-influenza viruses have on hospital admissions for adults will aid in assessing the expected population health benefits of influenza vaccines, and for any future vaccines and treatments for other respiratory viruses. This is particularly important in our aging population with an increasing prevalence of chronic diseases, as the impacts of respiratory virus epidemics will likely become more severe as the population ages [15].

To the best of our knowledge, no study has investigated the frequency and severity of influenza subtypes as well as a broad range of other respiratory viruses in adults hospitalised in Australia. This information is important in defining the health burden of all respiratory viruses, not only on the frequency of these admissions, but also for the clinical outcomes and the level of care required for these patients.

The aim of this study was to investigate the role of different respiratory viruses in the severity of outcomes among patients admitted to an adult tertiary teaching hospital in Perth, Western Australia.

## Methods

This was a retrospective cohort study analysing data from electronic records of patients admitted to Sir Charles Gairdner Hospital (SCGH) from January 2012 to December 2015. SCGH is a large adult tertiary teaching hospital with over 600 beds located in Perth, Western Australia. It treats approximately 93 000 inpatients and 60 000 Emergency Department presentations annually [16]. Patients aged 18 years and above who were admitted to SCGH with a respiratory illness were included in this study. The hospital information system data from that time period were searched using the following codes from the International Classification of Diseases, Tenth Revision, Clinical Modification (ICD-10-CM) to capture all patients presenting with a possible respiratory illness: J00–J06 (acute upper respiratory infections), J09–J18 (influenza and pneumonia), J20–J22 (other acute lower respiratory infections), J40–J47 (chronic lower respiratory diseases), J80 (acute respiratory distress syndrome) and J81 (pulmonary oedema). ICD-10-CM codes were selected to capture all patients who may have received a respiratory viral test. Data from patients meeting the search criteria in their primary diagnosis code, or in up to four further diagnosis/complication codes, were retrieved from the information system. These codes were also used to determine if patients had comorbidities such as chronic obstructive pulmonary disease (COPD), cardiovascular disease (CVD), diabetes, compromised immunity, asthma or cystic fibrosis. Although pregnancy and obesity are recognised risk factors for severe influenza, they were not included in this study due to very low numbers of patients identified through the ICD-10 codes. Available patient data also included patient demographics, admission and discharge dates, discharge codes and number of hours spent in ICU or on ventilation.

Virological testing data were obtained from PathWest Laboratory Medicine WA (PathWest), who perform routine virological testing for SCGH. During the study period samples were tested for A/H1, A/H3, INF-B, PIV and RSV using a tandem multiplex reverse transcription polymerase chain reaction using primers and probes from a previously described method [17], with an additional target for the hMPV matrix gene [18].

An SCGH patient record was matched with a PathWest record if the patient received virological testing within 7 days either side of their hospital admission date. Records were matched using either Unit Medical Record Number and date of birth, or name and date of birth. Any patients with multiple samples taken during the same admission were matched to the first sample collected that was positive for a virus. Multiple admissions occurring within 7 days of the first admission date were treated as a single admission for this study, otherwise they were analysed as separate admissions.

### Data analysis

Data analysis was undertaken using STATA 13 (College Station, Texas, USA). Age was categorised into three categories (18–49 years, 51–64 years and 65 plus) to reflect current Australian recommendations for influenza vaccination [19]. For univariate analysis of categorical variables, relative risks and 95% confidence intervals (CI) were calculated and *χ*^2^ or Fisher's exact test were used where appropriate to assess statistical significance of relative risks. We used the Students *t* test or Mann–Whitney *U* test to assess statistical significance of differences in continuous variables between groups. Severity was compared between A/H1, A/H3 and the other viruses using seven outcome variables: diagnosis of pneumonia, admission to ICU, requirement for assisted ventilation, time to discharge, time in ICU, time on assisted ventilation and death. Multivariable models (logistic or Cox regression) were constructed for each outcome, comparing patients with INF-B, RSV, hMPV or PIV to those with A/H1 or A/H3. For the categorical outcome variables of ICU admission, ventilation and pneumonia (all yes/no), logistic regression was used. We used Cox Proportional Hazards Regression to assess differences in the risk of discharge between patients with different viral infections at any particular point in time, as an indicator of length of hospitalisation. Deceased patients were excluded from the Cox regression models. Patients with no virus detected, multiple viruses detected or influenza A unsubtyped, were excluded in all multivariable models. A *P*-value less than 0.05 was considered significant for all tests. The following variables were included in the initial model: virus type, age group, sex, COPD, CVD, diabetes, immunocompromised, asthma and cystic fibrosis. Variables were removed consecutively from the model in order of descending *P*-values, so that they were only retained if they were significant (*P* < 0.05) or showed evidence of confounding, with the exception of sex and age group which were retained for all models. A variable was determined to be a confounder if the difference between the adjusted and unadjusted OR was greater than 15%.

### Ethics

Ethics approval was granted by SCGH and Curtin University Human Research Ethics Committees (Approval numbers 2016-058 and 10492 respectively).

## Results

### Viruses

There were 17 332 cases of possible respiratory illness admitted to SCGH from January 2012 to December 2015. Of these, 1753 (10.1%) were tested for respiratory viruses. Patients who were tested were younger than patients who were not tested (median 60 y.o. *vs.* 67 y.o., *P* < 0.001). There was no significant difference in the presence of comorbidities between patients who were and were not tested (50.1% *vs.* 49.1% respectively, *P* = 0.23), however those that were tested were more likely to have been admitted to ICU than those not tested (18.7% *vs.* 7.4%, *P* < 0.001).

Of the patients who were tested, 563 (32.1%) tested positive for at least one virus (Table 1). A/H3 was the most common, being detected in 177 (10.1%) patients. The median age of patients with a virus detected was 65 years. Patients with A/H1 were younger than patients with A/H3 (*χ*^2^ = 27.0, *P* < 0.001), INF-B (*χ*^2^ = 8.78, *P* = 0.012), RSV (*χ*^2^ = 17.66, *P* < 0.001), hMPV (*χ*^2^ = 10.65, *P* = 0.005) or PIV (*χ*^2^ = 15.79, *P* < 0.001) with only 21.6% of patients being over 65 years (Table 1), compared to between 47.6% and 58.8% for the other viruses. Eleven patients (0.6%) had two viruses detected and three patients had an influenza A virus which could not be subtyped.

**Table 1.** Demographics and comorbidities of patients in the study

	Total	Any virus detected	No virus detected	A/H1	A/H3	INF-B	RSV	hMPV	PIV
Cases	1753	563 (32.1%)	1190 (67.9%)	65 (3.7%)	177 (10.1%)	77 (4.4%)	101 (5.8%)	84 (4.8%)	68 (3.9%)
Age group									
18–49 y.o.	496 (28.3%)	131 (23.3%)	365 (30.7%)	23 (35.4%)	40 (22.7%)	23 (29.9%)	19 (18.8%)	21 (25.0%)	8 (11.8%)
50–64 y.o.	442 (25.2%)	149 (26.5%)	293 (24.6%)	28 (43.1%)	33 (18.8%)	17 (22.1%)	25 (24.8%)	23 (27.4%)	24 (35.3%)
65 + y.o.	815 (46.5%)	283 (50.3%)	532 (44.7%)	14 (21.6%)*	104 (58.8%)^	37 (48.1%)^	57 (56.4%)^	40 (47.6%)^	36 (52.9%)^
Female	845 (48.2%)	279 (49.6%)	566 (47.6%)	26 (40.0%)	90 (50.8%)	40 (52.0%)	58 (57.4%)	40 (47.6%)	34 (50.0%)
Comorbidities	878 (50.1%)	276 (49.0%)	602 (50.6%)	29 (44.6%)	75 (42.4%)	33 (42.9%)	61 (60.4%)*	50 (59.5%)*	34 (50.0%)
COPD	324 (18.5%)	96 (17.1%)	228 (19.1%)	9 (13.9%)	24 (13.6%)	10 (13.0%)	29 (28.7%)*^	11 (13.1%)	15 (22.1%)
CVD	172 (9.8%)	63 (11.2%)	109 (9.2%)	9 (13.9%)	17 (9.6%)	8 (10.4%)	14 (13.9%)	9 (10.7%)	7 (10.3%)
Diabetes	193 (11.0%)	72 (12.8%)	121 (10.2%)	6 (9.2%)	24 (13.6%)	9 (11.7%)	8 (7.9%)	18 (21.4%)	7 (10.3%)
Immuno compromised	184 (10.5%)	50 (8.9%)	134 (11.3%)	7 (10.8%)	11 (6.2%)	3 (3.9%)	10 (9.9%)	14 (16.7%)*	5 (7.4%)
Asthma	131 (7.5%)	44 (7.8%)	87 (7.3%)	2 (3.1%)	15 (8.5%)	3 (3.9%)	11 (10.9%)	12 (14.3%)^	5 (7.4%)
Cystic fibrosis	30 (1.7%)	5 (0.9%)	25 (2.1%)	1 (1.5%)	1 (0.6%)	2 (2.6%)	0 (0%)	0 (0%)	0 (0%)

*Statistically significant difference compared to A/H3 in univariate analysis. ^Statistically significant difference compared to A/H1 in univariate analysis.

Of the 563 patients with a virus, 526 (93.4%) were tested either prior to, or within 2 days of hospital admission, indicating detection of possible nosocomial cases to be uncommon.

### Comorbidities

Comorbidities were present in approximately half of the hospitalised patients, and this did not differ significantly between those with or without a virus detected (49.1% and 50.6% respectively, *P* = 0.575). The commonest comorbidities amongst the patients who tested positive for one of these viruses were COPD in 17.1%, and diabetes in 12.8% (Table 1). Compared with patients with A/H3, comorbidities were more common in those who had hMPV (59.5% *vs.* 42.4%, RR 1.44, 95% CI 1.12–1.85, *P* = 0.004) or RSV (60.4% *vs.* 42.4%, RR 1.42, 95% CI 1.11–1.80, *P* = 0.005). A higher proportion of admitted patients with hMPV had compromised immune function (RR 2.75, 95% CI 1.31–5.79, *P* = 0.008) compared to admitted patients with A/H3 and a higher proportion had asthma (RR 7.87, 95% CI 1.04–59.99, *P* = 0.046) compared to patients with A/H1. A higher proportion of admitted patients with RSV had COPD compared to admitted patients with A/H3 or A/H1 (Table 1; RR 2.14, 95% CI 1.30–3.51, *P* = 0.003 and RR 1.99 95% CI 1.00–3.94, *P* = 0.049 respectively).

### Changes over time

The proportion of patients with a virus detected ranged from 26% in 2013 to 37.8% in 2012. The proportion of tested patients who were positive for A/H3 varied more over the four-year study period compared to other viruses, ranging from 7.1% in 2014 to 18.1% in 2012. A/H3 was the dominant influenza virus in two out of the four years studied (2012 and 2013) and was equally dominant with INF-B in 2015 (40 detections each, 7.7%), while A/H1 dominated in 2014. RSV detections were also prominent in 2015, equalling the number of A/H3 and INF-B detections. The proportion of patients testing positive for INF-B varied from 1.5% in 2014 to 7.7% in 2015 and for RSV from 4.2% in 2014 to 7.7% in 2015. The proportion of patients testing positive for hMPV and PIV did not vary much over time, from 3.1% in 2014 to 6.0% in 2012 and 3.1% in 2013 to 5.0% in 2015 respectively.

### Comparisons of severity measures

Patients with A/H1 had a higher risk of being admitted to ICU (RR 2.74 95% CI 1.52–4.93, *P* = 0.001), requiring ventilation (RR 2.50, 95% CI 1.11–5.59, *P* = 0.026) or developing pneumonia (RR 1.68 95% CI 1.27–2.23, *P* < 0.001) than did patients with A/H3. These patients also had longer hospitalisation time than patients with A/H3 (log rank *χ*^2^ = 7.61, *P* = 0.0058; Table 2). These differences remained significant in multivariable analyses for ICU admission (OR 2.5461, 95% CI 1.188–5.520, *P* = 0.016; Table 7), pneumonia (OR 3.042, 95% CI 1.632–5.673, *P* < 0.001; Table 8) and risk of discharge (HR 0.643, 95% CI 0.471–0.878, *P* = 0.005; Table 9).

**Table 2.** Clinical outcomes of patients in the study

	All tested patients	Any virus detected	No virus detected	A/H1	A/H3	INF-B	RSV	hMPV	PIV
LOS (days) Median (IQR)	5 (2–9)	4 (2–8)	5 (3–11)	5 (3–8)*	4 (2–7)^	3 (2–6)^	5 (3–18)*	4 (2–7)^	3 (2–9)
ICU admission	325 (18.6%)	68 (12.1%)	257 (21.6%)	19 (29.2%)*	18 (10.2%)^	8 (10.4%)^	11 (10.9%)^	5 (6.0%)^	7 (10.3%)^
Ventilation	209 (11.9%)	38 (6.8%)	171 (14.4%)	10 (15.4%)*	11 (6.2%)^	6 (7.8%)	5 (5.0%)^	1 (1.2%)^	4 (5.9%)
ICU (hours) Median (IQR)	120 (56–241)	115 (52–265)	120 (56–240)	97 (40–502)	119 (48–256)	201 (47.5–360)	120 (61–136)	111 (97–152)	97 (62–236)
Ventilation (hours) Median (IQR)	145 (86–262)	165 (91–467)	139 (83–240)	438.5 (147–759)	141 (90–358)	285 (91–379)	129 (102–159)	75 (75–75)	157.5 (96.5–173.5)
Pneumonia	775 (44.2%)	234 (41.6%)	541 (45.5%)	40 (61.5%)*	64 (36.2%)^	23 (29.9%)^	42 (41.6%)^	35 (41.7%)^	22 (48.5%)
Deceased	89 (5.1%)	14 (2.5%)	75 (6.3%)	2 (3.1%)	5 (2.8%)	3 (3.9%)	3 (3.0%)	1 (1.2%)	0 (0%)

*Statistically significant difference compared to A/H3 in univariate analysis. ^Statistically significant difference compared to A/H1 in univariate analysis.

Similarly, compared with A/H1 many of the non-influenza A viruses had less severe outcomes for ICU admission, ventilation or pneumonia (Table 2). In multivariable analyses, patients with INF-B, hMPV, PIV or RSV were less likely to be admitted to ICU than patients with A/H1 (OR 0.385, 95% CI 0.151–0.984, *P* = 0.046; OR 0.158 95% CI 0.049–0.507, *P* = 0.002; OR 0.364, 95% CI 0.135–0.979, *P* = 0.045; OR 0.389, 95% CI 0.162–0.936, *P* = 0.035 respectively; Table 3). Patients with hMPV had lower odds of ventilation than patients with A/H1 (OR 0.080 95% CI 0.010–0.647, *P* = 0.018; Table 4). Patients with INF-B or RSV had lower odds of pneumonia than patients with A/H1 (OR 0.269, 95% CI 0.130–0.557, *P* < 0.001; OR 0.483, 95% CI 0.246–0.951, *P* = 0.035; Table 5). Additionally, patients with INF-B, hMPV or PIV had a higher risk of discharge than patients with A/H1 (HR 1.620, 95% CI 1.129–2.325, *P* = 0.009; HR 1.497, 95% 1.055–2.124, *P* = 0.024; HR 1.533, 95% CI 1.069–2.198, *P* = 0.020; Table 6), indicating shorter lengths of hospitalisation.

**Table 3.** Multivariable logistic regression model for ICU admission (viruses compared to A/H1) *N* = 549

Outcome variable	Independent variable (IV; *n* = number of patients with IV in ICU/number of patients with IV in model)	Unadjusted OR (95% CI)	Unadjusted *P*-value	Adjusted OR (95% CI)	Adjusted *P*-value
ICU admission (*n* = 66)	Virus (reference: A/H1) (*n* = 18/63)				
	A/H3 (*n* = 18/173)	0.290 (0.140–0.604)	0.001	0.390 (0.181–0.842)	0.016
	INF-B (*n* = 8/72)	0.312 (0.125–0.781)	0.013	0.385 (0.151–0.984)	0.046
	hMPV (*n* = 4/80)	0.132 (0.042–0.413)	0.001	0.158 (0.049–0.507)	0.002
	PIV (*n* = 7/66)	0.297 (0.114–0.771)	0.013	0.364 (0.135–0.979)	0.045
	RSV (*n* = 11/95)	0.327 (0.142–0.753)	0.009	0.389 (0.162–0.936)	0.035
	COPD (*n* = 18/92)	1.311 (0.975–7.763)	0.073	2.762 (1.438–5.306)	0.002
	Female (*n* = 26/269)	0.635 (0.496–0.812)	<0.001	0.635 (0.367–1.098)	0.104
	Age group (reference: 18–49 years, *n* = 22/127)				
	50–64 years (*n* = 23/146)	0.968 (0.714–1.313)	0.836	0.794 (0.404–1.560)	0.503
	65+ years (*n* = 21/276)	0.495 (0.370–0.663)	<0.001	0.356 (0.177–0.715)	0.004

**Table 4.** Multivariable logistic regression model for ventilation (viruses compared to A/H1) *N* = 549

Outcome variable	Independent variable (IV; *n* = number of patients with IV ventilated/number of patients with IV in model)	Unadjusted OR (95% CI)	Unadjusted *P*-value	Adjusted OR (95% CI)	Adjusted *P*-value
Ventilation (*n* = 37)	Virus (reference: A/H1) (*n* = 10/63)				
	A/H3 (*n* = 11/173)	0.360 (0.145–0.894)	0.028	0.464 (0.180–1.197)	0.112
	INF-B (*n* = 6/72)	0.481 (0.164–1.411)	0.183	0.562 (0.189–1.674)	0.301
	hMPV (*n* = 1/80)	0.067 (0.008–0.540)	0.011	0.080 (0.010–0.647)	0.018
	PIV (*n* = 4/66)	0.342 (0.101–1.154)	0.084	0.426 (0.123–1.473)	0.178
	RSV (*n* = 5/95)	0.294 (0.096–0.908)	0.033	0.379 (0.120–1.199)	0.099
	Female (*n* = 15/269)	0.667 (0.497–0.896)	0.007	0.709 (0.355–1.417)	0.330
	Age group (reference: 18–49 years, *n* = 12/127)				
	50–64 years (*n* = 13/146)	0.939 (0.657–1.341)	0.729	0.969 (0.416–2.257)	0.942
	65+ years (*n* = 12/276)	0.465 (0.327–0.660)	<0.001	0.506 (0.214–1.195)	0.120

**Table 5.** Multivariable logistic regression model for pneumonia (viruses compared to A/H1) *N* = 549

Outcome variable	Independent variable (IV; *n* = number of patients with IV and pneumonia/number of patients with IV in model)	Unadjusted OR (95% CI)	Unadjusted *P*-value	Adjusted OR (95% CI)	Adjusted *P*-value
Pneumonia (*n* = 230)	Virus (reference: A/H1) (*n* = 38/63)				
	A/H3 (*n* = 62/173)	0.367 (0.203–0.665)	0.001	0.329 (0.176–0.613)	<0.001
	INF-B (*n* = 23/72)	0.309 (0.152–0.626)	0.001	0.269 (0.130–0.557)	<0.001
	hMPV (*n* = 34/80)	0.486 (0.248–0.952)	0.035	0.531 (0.264–1.067)	0.075
	PIV (*n* = 32/66)	0.619 (0.308–1.25)	0.179	0.592 (0.287–1.220)	0.155
	RSV (*n* = 41/95)	0.500 (0.261–0.954)	0.036	0.483 (0.246–0.951)	0.035
	Immunocompromised (*n* = 14/50)	0.407 (0.288–0.573)	<0.001	0.422 (0.218–0.817)	0.011
	Asthma (*n* = 7/41)	0.297 (0.191–0.461)	<0.001	0.249 (0.106–0.582)	0.001
	Female (*n* = 114/269)	0.849 (0.703–1.027)	0.091	1.167 (0.819–1.663)	0.392
	Age group (reference: 18–49 years, *n* = 52/127)				
	50–64 years (*n* = 57 146)	0.936 (1.722–1.214)	0.619	0.849 (0.509–1.416)	0.531
	65+ years (*n* = 121/276)	1.141 (0.911–1.430)	0.250	1.164 (0.740–1.830)	0.511

**Table 6.** Cox regression model for probability of discharge from hospital over time (viruses compared to A/H1) *N* = 535

Outcome variable	Independent variable	Unadjusted HR (95% CI)	Unadjusted *P*-value	Adjusted HR (95% CI)	Adjusted *P*-value
Discharge from hospital (*n* = 535)	Virus (reference: A/H1) (*n* = 61)				
	A/H3 (*n* = 168)	1.591 (1.170–2.162)	0.003	1.555 (1.139–2.123)	0.005
	INF-B (*n* = 69)	1.681 (1.174–2.407)	0.005	1.620 (1.129–2.325)	0.009
	hMPV (*n* = 79)	1.539 (1.086–2.181)	0.015	1.497 (1.055–2.124)	0.024
	PIV (*n* = 66)	1.419 (0.982–2.051)	0.062	1.533 (1.069–2.198)	0.020
	RSV (*n* = 92)	1.196 (0.851–1.680)	0.301	1.263 (0.898–1.775)	0.180
	COPD (*n* = 89)	0.919 (0.805–1.049)	0.210	0.765 (0.605–0.969)	0.027
	Female (*n* = 264)	1.189 (1.079–1.310)	<0.001	0.942 (0.792–1.120)	0.496
	Age group (reference: 18–49 years, *n* = 125)				
	50–64 years (*n* = 142)	0.838 (0.735–0.956)	0.009	0.815 (0.637–1.043)	0.104
	65+ years (*n* = 268)	0.980 (0.873–1.099)	0.725	0.836 (0.668–1.045)	0.116

When compared to A/H3, the non-influenza A viruses did not show any statistical differences in severity outcomes (Tables 7–9), with the possible exception of PIV which, in patients with pneumonia, had borderline higher odds of being detected than A/H3 (OR 1.801 95% CI 0.999–3.49, *P* = 0.051) in multivariable analysis (Table 8).

**Table 7.** Multivariable logistic regression model for ICU admission (viruses compared to A/H3) *N* = 549

Outcome variable	Independent variable (IV; *n* = number of patients with IV in ICU/number of patients with IV in model)	Unadjusted OR (95% CI)	Unadjusted *P*-value	Adjusted OR (95% CI)	Adjusted *P*-value
ICU admission (*n* = 66)	Virus (reference: A/H3) (*n* = 18/173)				
	A/H1 (*n* = 18/63)	0.344 (1.655–7.167)	0.001	2.561 (1.188–5.520)	0.016
	INF-B (*n* = 8/72)	1.076 (0.445–2.601)	0.870	0.986 (0.401–2.425)	0.975
	hMPV (*n* = 4/80)	0.453 (0.148–1.386)	0.165	0.405 (0.131–1.259)	0.118
	PIV (*n* = 7/66)	1.021 (0.406–2.571)	0.964	0.931 (0.360–2.406)	0.883
	RSV (*n* = 11/95)	1.128 (0.509–2.500)	0.767	0.997 (0.439–2.261)	0.994
	COPD (*n* = 18/92)	1.311 (0.975–7.763)	0.073	2.762 (1.438–5.306)	0.002
	Female (*n* = 26/269)	0.635 (0.496–0.812)	<0.001	0.635 (0.367–1.098)	0.104
	Age group (reference: 18–49 years, *n* = 22/127)				
	50–64 years (*n* = 23/146)	0.968 (0.714–1.313)	0.836	0.794 (0.404–1.560)	0.503
	65+ years (*n* = 21/276)	0.495 (0.370–0.663)	<0.001	0.356 (0.177–0.715)	0.004

**Table 8.** Multivariable logistic regression model for pneumonia (viruses compared to A/H3) *N* = 549

Outcome variable	Independent variable (IV; *n* = number of patients with IV and pneumonia/number of patients with IV in model)	Unadjusted OR (95% CI)	Unadjusted *P*-value	Adjusted OR (95% CI)	Adjusted *P*-value
Pneumonia (*n* = 230)	Virus (reference: A/H3) (*n* = 62/173)				
	A/H1 (*n* = 38/63)	2.721 (1.504–4.923)	0.001	3.042 (1.632–5.673)	<0.001
	INF-B (*n* = 23/72)	0.840 (0.468–1.508)	0.560	0.819 (0.452–1.485)	0.512
	hMPV (*n* = 34/80)	1.323 (0.770–2.274)	0.311	1.616 (0.920–2.838)	0.095
	PIV (*n* = 32/66)	1.685 (0.949–2.991)	0.075	1.801 (0.999–3.249)	0.051
	RSV (*n* = 41/95)	1.359 (0.815–2.266)	0.239	1.471 (0.871–2.485)	0.149
	Immunocompromised (*n* = 14/50)	0.407 (0.288–0.573)	<0.001	0.422 (0.218–0.817)	0.011
	Asthma (*n* = 7/41)	0.297 (0.191–0.461)	<0.001	0.249 (0.106–0.582)	0.001
	Female (*n* = 114/269)	0.849 (0.703–1.027)	0.091	1.167 (0.819–1.663)	0.392
	Age group (reference: 18–49 years, *n* = 52/127)				
	50–64 years (*n* = 57/146)	0.936 (1.722–1.214)	0.619	0.849 (0.509–1.416)	0.531
	65+ years (*n* = 121/276)	1.141 (0.911–1.430)	0.250	1.164 (0.740–1.830)	0.511

**Table 9.** Cox regression model for probability of discharge from hospital over time (viruses compared to A/H3) *N* = 535

Outcome variable	Independent variable	Unadjusted HR (95% CI)	Unadjusted *P*-value	Adjusted HR (95% CI)	Adjusted *P*-value
Discharge from hospital (*n* = 535)	Virus (reference: A/H3) (*n* = 168)				
	A/H1 (*n* = 61)	0.629 (0.463–0.854)	0.003	0.643 (0.471–0.878)	0.005
	INF-B (*n* = 69)	1.057 (0.795–1.405)	0.704	1.042 (0.785–1.384)	0.776
	hMPV (*n* = 79)	0.968 (0.738–1.268)	0.811	0.963 (0.736–1.259)	0.781
	PIV (*n* = 66)	0.892 (0.663–1.200)	0.451	0.986 (0.740–1.313)	0.922
	RSV (*n* = 92)	0.752 (0.579–0.976)	0.032	0.812 (0.626–1.053)	0.116
	COPD (*n* = 89)	0.919 (0.805–1.049)	0.210	0.765 (0.605–0.969)	0.027
	Female (*n* = 264)	1.189 (1.079–1.310)	<0.001	0.942 (0.792–1.120)	0.496
	Age group (reference: 18–49 years, *n* = 125)				
	50–64 years (*n* = 142)	0.838 (0.735–0.956)	0.009	0.815 (0.637–1.043)	0.104
	65+ years (*n* = 268)	0.980 (0.873–1.099)	0.725	0.836 (0.668–1.045)	0.116

## Discussion

This study is one of the few to undertake a detailed comparison of admissions and clinical outcomes of the individual influenza virus types and subtypes and the non-influenza viruses in hospitalised adults. We used data retrieved from electronic health records to identify the respiratory viruses found in adults admitted to a tertiary hospital in Australia during the period 2012–2015, and used influenza A viruses as a comparator to assess the severity of illness due to the other respiratory viruses.

Overall A/H3 was the most frequently detected virus in adults admitted to our hospital for a respiratory illness. Furthermore, either A/H3 or A/H1 was the dominant or equally dominant virus in every year of the study. That is consistent with the documented major role of influenza A viruses as causes of more severe influenza illness in adults, especially the elderly [20]. However, across the study period, the total patients with non-influenza A viruses outnumbered those with influenza A viruses, and the numbers of hospitalised cases with RSV and INF-B were equal to that of A/H3 in 2015. Previous studies investigating the number of hospitalisations for non-influenza respiratory viruses are conflicting, with some studies showing the number of hospitalisations for influenza are greater than for RSV or hMPV [11, 12, 21–23], while others found that respiratory hospitalisations for RSV and/or hMPV are similar to influenza [24–27]. This may vary according to the circulating influenza strains in each season.

Patients infected with A/H1 had more severe clinical outcomes than those infected with A/H3, INF-B, RSV or hMPV. However, patients with A/H3 had similar clinical outcomes to the non-influenza viruses. This indicates that these viruses contribute substantially to the healthcare burden of respiratory viruses. Previous research has shown that for patients infected with respiratory viruses other than influenza who are admitted to hospital, the outcomes can be just as severe as for those with influenza, with similar rates of ICU admission, ventilation and death [13]. Hospitalised RSV cases in particular have similar or greater odds of pneumonia, ICU admission, ventilation and death compared to influenza [13, 14, 25, 26, 28].

We showed that patients with A/H1 were more likely to be admitted to ICU, develop pneumonia and be hospitalised longer than patients with A/H3, indicating that A/H1 is more severe than A/H3 in this population, despite this virus affecting younger ages (mean age 53 *vs.* 65 years for H1 and H3 patients respectively). In 2015, patients with A/H1 outnumbered those with A/H3 in ICU admissions, despite having nearly 80% fewer hospitalisations (four ICU admissions from nine A/H1 admissions *vs.* 3/40 H3 admissions). This is consistent with previous studies that have shown that A/H1 has, since 2009, remained the dominant cause of clinically severe influenza, particularly in younger adults [3–6, 29], even though A/H3 was the dominant subtype causing hospital admission. In previous influenza pandemics, the pandemic strain has been associated with a high proportion of deaths in patients under 65 years, which continues, albeit at lower proportions, in the decade following the pandemic [30]. Our study provides further evidence that post-pandemic influenza can still cause severe disease in young adults. However, in other populations A/H3 has been a larger contributor to severe illness [31], but this can vary with age [32, 33], so the impact of influenza A will differ from season-to-season [34, 35]. In our multivariable analyses we adjusted for age, and therefore this impact may not be apparent.

Overall, comorbidities were common, being present in approximately half of the admitted patients. However, the presence of comorbidity was significantly less likely in patients with A/H3, compared to those with RSV or hMPV. Co-morbidities were also less frequently present in patients with A/H1, however statistical significance was not reached (*P* = 0.074 and 0.067 for hMPV and RSV respectively). The lower number of comorbidities in patients with influenza may be due to a high proportion of people with comorbidities receiving the annual influenza vaccine, but vaccination status for the patients in our study was not available. Another possible explanation for our findings is that influenza A is capable of causing hospitalisation in relatively healthy people, whereas the other viruses may cause less severe disease in patients without comorbidities.

This study had a number of limitations. As this was a retrospective study, we could not investigate the impacts of viruses that were not routinely tested for. Some patients received additional testing for other respiratory viruses such as adenovirus, rhinovirus and coronavirus, but as the number of patients with these tests was low, and there may be a selection bias for those who received extra testing, these were not included in the study. Rhinovirus in particular has been implicated in previous studies as a possible cause of severe respiratory disease, having been detected in a large proportion of hospitalised patients [11, 21, 22, 27]. In addition it has also been shown that influenza A and rhinovirus infections have similar measures of severity in immunocompromised patients [36].

This study did not take into account the effect of influenza vaccination on our population. In 2015, influenza vaccination coverage rates in Australian hospitalised patients with acute respiratory illness was estimated to be 80.2% in patients aged over 65, and 57.9% in adults under 65 with comorbidities, with the proportion of patients requiring ICU being slightly lower in the vaccinated group than the unvaccinated group (5.7% *vs.* 8.6%) [37]. Influenza vaccine effectiveness against hospitalisation in the elderly and people with comorbidities varies according to the matching of the vaccine strains with circulating strains [38]. While we found that non-influenza respiratory viruses can cause just as severe disease like influenza, we cannot say if this would be the case in an unvaccinated population. It is possible that influenza vaccination reduced both the frequency and severity of influenza hospitalisations, therefore this should be taken into account when interpreting the results of this study. We did not have information about the influenza vaccination status or antiviral treatment for the cases in our study. Therefore we are unable to assess whether either treatment or vaccination attenuated the clinical picture of influenza cases. In addition, while attempts were made to minimise detections of nosocomial influenza, the presence of nosocomial cases in our study cannot be excluded.

The study also did not consider the overall prevalence of influenza A viruses in the broader population during the study period. Therefore, we are unable to determine whether the proportion of hospitalised cases with the different influenza A strains reflected the circulation of these strains in the population or severity of infection with each strain. Also the relative impact of influenza *vs.* the other respiratory viruses will vary across time depending on the comparative levels of activity of the different viruses with the community.

While our study involved 17 332 hospital admissions, the proportion of patients tested for respiratory viruses was small (10%). The selection of ICD-10-CM codes used in this study to identify patients with respiratory illness was broad, which may account for the small proportion of testing. In addition, pathology testing for respiratory viruses was proportionally higher in winter compared to the non-winter months in our study (data not shown). Infections during the non-winter months may have been missed due to lower rates of testing [39]. In our study the rate of ICU admission among tested patients was higher than that of untested patients, therefore we cannot discount that a selection bias may exist for testing of severely ill patients which could skew results. In addition insufficient power may have been a limitation of this study. Additional studies are needed to further investigate the level of severity of disease in hospitalised cases with non-influenza respiratory virus infections.

We have found that hospitalised patients with influenza A/H1 had more severe clinical outcomes than patients with A/H3, INF-B and the non-influenza respiratory viruses. Knowledge of the influenza strains circulating and their associated clinical outcomes will aid in hospital preparedness during the influenza season. Future research is needed to investigate the impact of a broader range of viruses associated with respiratory hospitalisations.

## Supplementary material

For supplementary material accompanying this paper visit http://dx.doi.org/10.1017/S095026881900195X. click here to view supplementary material
