# Supplementary material for: Post-pandemic influenza A/H1N1pdm09 is associated with more severe outcomes than A/H3N2 and other respiratory viruses in adult hospitalisations
Source: Epidemiol Infect. 2019 Nov 28;147:e310. doi: 10.1017/S095026881900195X (PMC7003621; doi:10.1017/S095026881900195X)
Supplement: Supplementary file 1 [file S095026881900195Xsup001.docx]

**APPENDIX A:** ICD-10 codes used to distinguish pneumonia and co-morbidities. All codes were decided upon by the author upon examination of the ICD-10 codes, with the exception of the immunocompromised codes which were used from U.S Department of Health & Human Services (2015).

| **Illness** | **ICD-10 Codes** |
| --- | --- |
| Pneumonia | J09.X1, J10.0, J10.00, J10.01, J10.08, J11.0, J11.00, J11.08 J12., J12.0, J12.1, J12.2, J12.3, J12.8, J12.81, J12.89, J12.9, J13., J14., J15., J15.0, J15.1, J15.2, J15.20, J15.21, J15.211, J15.212, J15.29, J15.3, J15.4, J15.5, J15.6, J15.7, J15.8, J15.9, J16., J16.0, J16.8, J17., J17.0, J18., J18.0, J18.1, J18.2, J18.8, J18.9 |
| COPD | J44, J44.0, J44.1, J44.9 |
| CVD | I10., I11., I11.0, I11.9, I13., I13.0, I13.1, I13.10, I15., I15.0, I15.1, I15.2, I15.8, I15.9, I20., I20.0, I20.1, I20.8, I20.9, I21., I21.0, I21.01, I21.02, I21.09, I21.1, I21.11, I21.19, I21.2, I21.21, I21.29, I21.3, I21.4, I22., I22.0, I22.1, I22.2, I22.8, I22.9, I23., I23.0, I23.1, I23.2, I23.3, I23.4, I23.5, I23.6, I23.7, I23.8, I24., I24.0, I24.1, I24.8, I24.9, I25., I25.1, I25.10, I25.11, I25.110, I25.111, I25.118, I25.119, I25.2, I25.3, I25.4, I25.41, I25.42, I25.5, I25.6, I25.7, I25.70, I25.700, I25.701, I25.708, I25.709, I25.71, I25.710, I25.711, I25.718, I25.719, I25.72, I25.720, I25.721, I25.728, I25.729, I25.73, I25.730, I25.731, I25.738, I25.739, I25.75, I25.750, I25.751, I25.758, I25.759, I25.76, I25.760, I25.761, I25.768, I25.769, I25.79, I25.790, I25.791, I25.798, I25.799, I25.8, I25.81, I25.810, I25.811, I25.812, I25.82, I25.83, I25.84, I25.89, I25.9, I26., I26.0, I26.01, I26.02, I26.09, I26.9, I26.90, I26.92, I26.99, I27., I27.0, I27.1, I27.2, I27.8, I27.81, I27.82, I27.89, I27.9, I28., I28.0, I28.1, I28.8, I28.9 |
| Diabetes | E08., E08.0, E08.00, E08.01, E08.1, E08.10, E08.11, E08.2, E08.21, E08.22, E08.29, E08.3, E08.31, E08.311, E08.319, E08.32, E08.321, E08.329, E08.33, E08.331, E08.339, E08.34, E08.341, E08.349, E08.35, E08.351, E08.359, E08.36, E08.39, E08.4, E08.40, E08.41, E08.42, E08.43, E08.44, E08.49, E08.5, E08.51, E08.52, E08.59, E08.6, E08.61, E08.610, E08.618, E08.62, E08.620, E08.621, E08.622, E08.628, E08.63, E08.630, E08.638, E08.64, E08.641, E08.649, E08.65, E08.69, E08.8, E08.9, E09., E09.0, E09.00, E09.01, E09.1, E09.10, E09.11, E09.2, E09.21, E09.22, E09.29, E09.3, E09.31, E09.311, E09.319, E09.32, E09.321, E09.329, E09.33, E09.331, E09.339, E09.34, E09.341, E09.349, E09.35, E09.351, E09.359, E09.36, E09.39, E09.4, E09.40, E09.41, E09.42, E09.43, E09.44, E09.49, E09.5, E09.51, E09.52, E09.59, E09.6, E09.61, E09.610, E09.618, E09.62, E09.620, E09.621, E09.622, E09.628, E09.63, E09.630, E09.638, E09.64, E09.641, E09.649, E09.65, E09.69, E09.8, E09.9, E10., E10.1, E10.10, E10.11, E10.2, E10.21, E10.22, E10.29, E10.3, E10.31, E10.311, E10.319, E10.32, E10.321, E10.329, E10.33, E10.331, E10.339, E10.34, E10.341, E10.349, E10.35, E10.351, E10.359, E10.36, E10.39, E10.4, E10.40, E10.41, E10.42, E10.43, E10.44, E10.49, E10.5, E10.51, E10.52, E10.59, E10.6, E10.61, E10.610, E10.618, E10.62, E10.620, E10.621, E10.622, E10.628, E10.63, E10.630, E10.638, E10.64, E10.641, E10.649, E10.65, E10.69, E10.8, E10.9, E11., E11.0, E11.00, E11.01, E11.2, E11.21, E11.22, E11.29, E11.3, E11.31, E11.311, E11.319, E11.32, E11.321, E11.329, E11.33, E11.331, E11.339, E11.34, E11.341, E11.349, E11.35, E11.351, E11.359, E11.36, E11.39, E11.4, E11.40, E11.41, E11.42, E11.43, E11.44, E11.49, E11.5, E11.51, E11.52, E11.59, E11.6, E11.61, E11.610, E11.618, E11.62, E11.620, E11.621, E11.622, E11.628, E11.63, E11.630, E11.638, E11.64, E11.641, E11.649, E11.65, E11.69, E11.8, E11.9, E13., E13.0, E13.00, E13.01, E13.1, E13.10, E13.11, E13.2, E13.21, E13.22, E13.29, E13.3, E13.31, E13.311, E13.319, E13.32, E13.321, E13.329, E13.33, E13.331, E13.339, E13.34, E13.341, E13.349, E13.35, E13.351, E13.359, E13.36, E13.39, E13.4, E13.40, E13.41, E13.42, E13.43, E13.44, E13.49, E13.5, E13.51, E13.52, E13.59, E13.6, E13.61, E13.610, E13.618, E13.62, E13.620, E13.621, E13.622, E13.628, E13.63, E13.630, E13.638, E13.64, E13.641, E13.649, E13.65, E13.69, E13.8, E13.9 |
| Immunocompromised | B20., B59., C80.2, C88.8, C94.40, C94.41, C94.42, C94.6, D47.1, D47.9, D47.Z1, D47.Z9, D61.1, D61.810, D61.811, D61.818, D70., D70.0, D70.1, D70.2, D70.4, D70.8, D70.9, D71., D72.0, D72.810, D72.818, D72.819, D73.81, D75.81, D76., D76.1, D76.2, D76.3, D80., D80.0, D80.1, D80.2, D80.3, D80.4, D80.5, D80.6, D80.7, D80.8, D80.9, D81., D81.0, D81.1, D81.2, D81.4, D81.6, D81.7, D81.89, D81.9, D82., D82.0, D82.1, D82.2, D82.3, D82.4, D82.8, D82.9, D83., D83.0, D83.1, D83.2, D83.8, D83.9, D84., D84.0, D84.1, D84.8, D84.9, D89.3, D89.8, D89.81, D89.810, D89.811, D89.812, D89.813, D89.82, D89.89, D89.9, E40., E41., E42., E43., I12.0, I13.11, I13.2, K91.2, M35.9, N18.5, N18.6, T86., T86.0, T86.00, T86.01, T86.02, T86.03, T86.09, T86.1, T86.10, T86.11, T86.12, T86.13, T86.19, T86.2, T86.20, T86.21, T86.22, T86.23, T86.29, T86.290, T86.298, T86.3, T86.30, T86.31, T86.32, T86.33, T86.39, T86.4, T86.40, T86.41, T86.42, T86.43, T86.49, T86.5, T86.8, T86.81, T86.810, T86.811, T86.812, T86.818, T86.819, T86.82, T86.820, T86.821, T86.822, T86.828, T86.829, T86.83, T86.830, T86.831, T86.832, T86.838, T86.839, T86.85, T86.850, T86.851, T86.85, T86.858, T86.859, T86.89, T86.890, T86.891, T86.892, T86.898, T86.899, T86.9, T86.90, T86.91, T86.92, T86.93, T86.99, Z48.2, Z48.21, Z48.22, Z48.23, Z48.24, Z48.28, Z48.280 , Z48.288, Z48.29, Z48.290, Z48.298, Z49.01, Z49.02, Z49.31, Z94., Z94.0, Z94.1, Z94.2, Z94.3, Z94.4, Z94.8, Z94.81, Z94.82, Z94.83, Z94.84, Z94.89, Z99.2 |
| Asthma | J45., J45.2, J45.20, J45.21, J45.22, J45.3, J45.30, J45.31, J45.32, J45.4, J45.40, J45.41, J45.42, J45.5, J45.50, J45.51, J45.52, J45.9, J45.90, J45.901, J45.902, J45.909, J45.99, J45.990 ,J45.991, J45.998 |
| Cystic Fibrosis | E84., E84.0, E84.1, E84.11, E84.19, E84.8, E84.9 |

*Table A1: ICD-10 codes used to distinguish pneumonia and co-morbidities*

U.S Department of Health & Human Services. (2015). Immunocompromised State Diagnosis and Procedure Codes. Retrieved from <http://www.qualityindicators.ahrq.gov/>
